# Children Conceived by Assisted Reproductive Technology Prone to Low Birth Weight, Preterm Birth, and Birth Defects: A Cohort Review of More Than 50,000 Live Births During 2011–2017 in Taiwan

**DOI:** 10.3389/fped.2020.00087

**Published:** 2020-03-13

**Authors:** Heng-Yu Chang, Wuh-Liang Hwu, Ching-Hui Chen, Chun-Yin Hou, Wei Cheng

**Affiliations:** ^1^Department of Biochemistry and Molecular Cell Biology, College of Medicine, Taipei Medical University, Taipei, Taiwan; ^2^Department of Medical Genetics, National Taiwan University Hospital, Taipei, Taiwan; ^3^Department of Obstetrics and Gynecology, Taipei Medical University Hospital, Taipei, Taiwan; ^4^Division of Reproductive Medicine, Department of Obstetrics and Gynecology, Taipei Medical University Hospital, Taipei, Taiwan; ^5^Department of Family Medicine, Taipei City Hospital, Taipei, Taiwan; ^6^Department of Pathology, Kee-Lung Hospital, Ministry of Health and Welfare, Keelung City, Taiwan; ^7^Department of Nursing, Ching Kuo Institute of Management and Health, Keelung City, Taiwan; ^8^School of Nursing, National Taipei University of Nursing and Health Sciences, Taipei, Taiwan

**Keywords:** assisted reproductive technology, lower birth weight, preterm birth, birth defects, live births

## Abstract

**Objectives:** The use of assisted reproductive technology (ART) has increased rapidly in Taiwan. The purpose of this study is to discuss the risks of low birth weight, preterm birth, and birth defect for children conceived by assisted reproductive technology in Taiwan.

**Methods:** Both National ART report database and National birth reports were obtained from the Health Promotion Administration in the Ministry of Health and Welfare in Taiwan. The cohort included live births (*n* = 1,405,625) and children conceived by ART (*n* = 50,988/172,818 cycles) from 2011 to 2017. The prevalence of low birth weight, preterm birth, and birth defect were compared between the ART and natural pregnancy groups.

**Results:** Children conceived by ART displayed a higher rate of low birth weight as compared to those in the natural pregnancy group (*p* < 0.001), even when analyses were restricted to singleton births (*p* < 0.001). A higher rate of preterm birth (*p* < 0.001) was also observed in children conceived by ART even when analyses were restricted to singleton births (*p* < 0.05). A significant increased rate of birth defects was noted from children conceived by ART *(p* < 0.05*)*.

**Conclusions:** With the increasing need for and use of ART-conceptions, the likelihood of risks induced or related to Assistant Reproductive Technology (ART) has drawn considerable attention in recent years. Taiwan, as one of the leading countries with outstanding ART performances and modern medical care, the result of the current study suggests that further consideration and tighter regulations and policy are needed with regard to the use of ART.

## Introduction

The first *in vitro* fertilization baby in Taiwan was born in 1985, and thereafter, the number of Assisted Reproductive Technology (ART) cycles has increased rapidly. The percentage of births by ART increased from 0.86% in 1998 to 1.44% in 2007, and then to 4.33% in 2016, with an annual increase of 41.23% (1). Possible social factors contributing to the increase of using ART are many; such as women postponing their childbearing age, busy jobs, higher social pressure, or even same-sex marriages. The government policy also supports the use of ART in the face of the decreasing birth rate in Taiwan. However, health risks associated with ART-conceptions have been reported in both animal studies and epidemiological surveys, which demands more attention and further considerations in future clinic practices.

Studies have revealed that ART increased the risks of preterm birth, low birth weight, congenital anomalies, and perinatal mortality as compared to that of natural pregnancies (2–6). The occurrences of preterm birth and low birth weight were still high in singleton births by ART (7). An increased incidence of preterm birth has been reported (8), and the risk could not be reduced even by elective single embryo transfer (9). Hansen et al. performed a meta-analysis of publications before 2003, and concluded that birth defects increased in children conceived by ART (10).

Sandin et al. studied more than 2.5 million children born in Sweden from 1982 through 2007 and found that the risk of mental retardation was significantly higher in children conceived by ART as compared to their counterparts through natural pregnancies (11). Fountain et al. studied 6 million live births and found that the incidence of autism was 2-fold as higher as in children conceived by ART than in natural pregnancy (12). Hansen et al. studied more than 210,000 children born in Western Australia between 1994 and 2002 and found an increased risk of intellectual disability for children conceived by ART (10). Researchers also reported an increase in arterial hypertension in children conceived by ART, probably due to ART-induced premature vascular aging (13, 14). Fetus conceived by ART also showed signs of cardiovascular remodeling, including a more globular heart, thick myocardial walls, decreased longitudinal functions, impaired relaxation, and dilated atria (15). An increased risks of cancer has also been observed in children conceived by ART (16–18).

In spite of the risks observed in children conceived by ART, the use of ART has increased rapidly in Taiwan. The aim of this study is to investigate the outcomes of children conceived by ART during 2011–2017 in Taiwan.

## Methods

### Data Sources

All the data of mothers and babies was obtained from a website-based reporting system designed by the Ministry of Health and Welfare in Taiwan and approved by the Ethics Committee of Taipei Hospital, Ministry of Health and Welfare (TH-IRB-0020-0002). Since 2007, the Ministry of Health and Welfare, MOHW (previously known as the Department of Health under the Executive Yuan) has requested all participating ART clinics and hospitals to register cycle-specific information for all ART treatment cycles. Such information includes patient characteristics, information on ART treatments, the pregnancy rate and obstetric outcomes. Both the National ART report database and National birth reports, which includes the yearly number of people by age and gender and national annual number of births, were obtained from the Health Promotion Administration in the Ministry of Health and Welfare. We therefore took advantage of this system to collaborate with MOHW, our cohort included natural pregnancy live births (*n* = 1,405,625) and children conceived by ART (*n* = 50,988/172,818 cycles) from 2011 to 2017.

### Measures and Statistical Analysis

We obtained and calculated the following variables: (1) rate of very low birth weight (VLBW, <1,500 g); number of VLBW by ART divided by national number of ART in the same year, (2) rate of low birth weight (LBW, 1,500–2,499 g); number of LBW by ART divided by national number of ART in the same year, (3) rate of preterm birth (gestational age <37 weeks); number of preterm births by ART divided by national number of ART in the same year, and (4) rate of birth defects (cardiovascular, central nervous system, gastrointestinal, urogenital, musculoskeletal, ear and facial and chromosome defects) by ART; number of birth defects by ART divided by national number of ART in the same year. We also obtained and calculated the same variables for the natural pregnancies and compared with those of ART conceptions. We applied a time series design for the measurements by *T*-Test.

## Results

### Demographic Characteristics of the Cohorts

The percentage of births by ART increased from 2.77% in 2011 to 4.92% in 2017 (Supplement Figure 1). More than 70% of pregnant women were younger than 34 years old, while 71.04% of women became pregnant through ART were older than 35 years old in 2017 (Table 1). The percentage of twin pregnancy was higher in ART conceptions than that in natural pregnancy, but the rates of twin birth in ART decrease slightly during the study period (Supplement Figure 2).

**Table 1:** Maternal age among total pregnancies and ART conceptions in 2017.

**Maternal age**	^*^Total pregnancies		**ART conceptions**	
	**(*****N*** **=** **195,113)**		**(cycles** **=** **37,849)**	
	**No**.	**%**	**No**.	**%**
≤ 24	18,965	9.71	129	0.3
25–34	119,496	61.23	10,830	28.6
≥35	56,652	29.03	26,890	71.04

* *The maternal age of natural pregnancies can't be obtained*.

### ART and Birth Weight

A total of 1,405,625 natural pregnancy births and 50,988 ART conception births from 2011 to 2017 were included in the analysis. The proportions of very low birth weight (4.12% vs. 0.76%, *p* < 0.001) and low birth weight (32.09% vs. 7.05%, *p* < 0.001) were significantly higher in ART conception than in natural pregnancy (Table 2). The differences in very low birth weight (1.94% vs. 0.76%, *p* < 0.001) and low birth weight (9.46% vs. 7.05%, *p* < 0.001) still hold true when the analyses were restricted to singletons only (Table 2). The annual proportions of very low birth weight and low birth weight in ART conception, ART conception singletons and natural pregnancy are in the Supplement Table 1.

**Table 2:** Proportion of very low birth weight (VLBW) and low birth weight (LBW) in natural pregnancy, ART conception and ART conception singleton in 2011–2017.

**Birthweight (g)**	**Natural pregnancy (*****N*** **=** **1,405,625)**		**ART conception (*****N*** **=** **50,988)**		***P-value***
	**No**.	**%**	**No**.	**%**	
VLBW (<1,500)	10,770	0.76	2,103	4.12	<0.001
LBW(1,500–2,499)	99,111	7.05	16,363	32.09	<0.001
**Birthweight (g)**	**Natural pregnancy (*****N*** **=** **1,405,625)**		**ART conception singleton (*****N*** **=** **28,455)**		***P-value***
	**No**.	**%**	**No**.	**%**	
VLBW (<1,500)	10,770	0.76	554	1.94	<0.001
LBW (1,500–2,499)	99,111	7.05	2,693	9.46	<0.001

### ART and Preterm Birth

A total of 611,200 natural pregnancy births and 22,078 ART conception births from 2014 to 2016 were included in the analysis. The preterm birth rate (<37 weeks) of ART conception was significantly higher than that of natural pregnancy (*p* < 0.001) (Table 3). The differences hold true when the analyses were restricted to singletons (Table 3). It is important to note, that rate of preterm birth in ART conception is higher in younger mothers (Table 3). The annual proportions of preterm births in ART conception, ART conception singletons and natural pregnancy are in the Supplement Table 2.

**Table 3:** Proportion of preterm birth (<37 weeks) in natural pregnancy, ART conception and ART conception singleton in 2014–2016.

**Maternal age**	**Natural pregnancy (*****N*** **=** **611,200)**		**ART conception (*****N*** **=** **22,078)**		***P-value***
	**No**.	**%**	**No**.	**%**	
≤ 24	5,002	8.3	53	48.6	<0.001
25–34	31,159	7.7	3,960	36.8	<0.001
≥35	15,348	10.4	3,756	33.5	<0.001
**Maternal age**	**Natural pregnancy (*****N*** **=** **611,200)**		**ART conception (*****N*** **=** **12,804)**		***P-value***
	**No**.	**%**	**No**.	**%**	
≤ 24	5,002	8.3	10	16.1	<0.05
25–34	31,159	7.7	650	11.3	<0.02
≥35	15,348	10.4	961	13.7	<0.01

### ART and Birth Defects

Infants born from 2014 to 2016 were included in this analysis. Children conceived by ART, compared to those through natural pregnancy, had an increased likelihood of developing any defects (0.83% vs. 0.42%, *p* < 0.05) (Table 4), cardiovascular defects (0.24% vs. 0.06%, *p* < 0.01) (item 1), central nervous system defects (0.06% vs. 0.01%, *p* < 0.05) (item 2), and gastrointestinal defects (0.09% vs. 0.03%, *p* < 0.05) (item 3). On the contrary, the percentage of urogenital, musculoskeletal, ear and facial defects and chromosome abnormality (items 4–7) were lower in children conceived by ART, probably because prenatal diagnoses and artificial abortions were routinely employed after ART conceptions. The annual proportions of birth defects in ART conception and natural pregnancy are in the Supplement Tables 3, 4.

**Table 4:** Prevalence of birth defects in Natural pregnancy and ART conception in 2014–2016.

^*^Birth defects	**Natural pregnancy (*****N*** **=** **611,200)**			**ART conception (*****N*** **=** **22,078)**			***P*-value**
	**No**.		**%**	**No**.		**%**	
Any defects	2,572		0.42	201		0.83	<0.05
1	385		0.06	58		0.24	<0.01
2	59		0.01	15		0.06	<0.05
3	205		0.03	22		0.09	<0.05
4	315		0.05	20		0.08	0.216
5	727		0.12	11		0.05	<0.001
6	724		0.12	7		0.03	<0.001
7	30		0.02	2		0.03	0.165

* *The labels of the defects: cardiovascular (1), central nervous system (2), gastrointestinal (3), urogenital (4), musculoskeletal (5), ear and facial defects (6), and chromosome abnormalities (7)*.

## Discussion

By reviewing the data of the recent cohort of ART-conceived children in Taiwan, we observed an increased occurrences of low birth weight, preterm birth, and birth defects. This study is unique that we analyzed a fairly large number (more than 50,000) of children who were conceived by ART during a recent period of time (from 2011 to 2017). Therefore, we were able to obtain highly significant results and to compare the prevalence of birth defects between the two groups. It is disappointing that complications still occurred in a country where outstanding ART technologies and modern medical care are in place.

Both embryo manipulation and environmental factors within the laboratory appear to cause epigenetic changes during the first stages of embryo development (19, 20). Alterations of gene expression occur (21), and these modifications can be heritable (22). The altered methylations of the endothelial nitric oxide synthase (eNOS) gene leads to decreased vascular NO synthesis in the aorta, and endothelial dysfunction and increased stiffness lead to arterial hypertension (23, 24). Exogenous Gonadotropins administered to the ovary during controlled ovarian stimulation causes epigenetic changes in peg1, kcnq1ot1, zac (Zac1), and h19 (25). *Zac1* plays an important role in neural stem cell quiescence, proliferation and differentiation (26). Either loss or gain of *Zac1* expression was reported to be associated with reduced growth rates and intellectual disability (27), and altered methylation of kcnq1ot1 and h19 was also reported in children with intellectual disability (28). Epigenetic impairments of DNA methylation were also related to pediatric pre-B cell acute lymphoblastic leukemia (29).

One limitation of the current study, similar to that of all these kinds of studies, is being unable to exclude the intrinsic risks of couples using ART, including the classic factors related to low birth weight, preterm birth, and birth defects. Generally, women undergoing ART are older and more often primiparous than the general obstetric population. Consequently, these mothers carry additional age and parity-related risks (30–32). Furthermore, the prevalence of chromosome abnormalities could not be accurately assessed in children conceived by ART because prenatal genetic diagnoses (PGD) and artificial abortions were routinely applied if the patients were desired while ART conceptions was performed in Taiwan.

## Conclusion

Taiwan has been well-known for its mature and outstanding ART technologies and modern medical care. However, the risks for children conceived by ART during 2011–2017 appeared to have increased, which suggests that the risks are more than a statistic result. Rather, it presents itself a real phenomenon that may or may not be avoided in ART procedures.

Thus, in order to reduce the risks in ART-conceived children, the current ART policy and regulations may consider revising granting permissions for ART applications, including extending the policy to suitable applicants only. For example, to reduce age-related risk factors, ART permission should not be granted to women aged 50 and over. It is also high time to educate public about risks of using ART and raise social awareness on this issue.

## Data Availability Statement

All datasets generated for this study are included in the article/Supplementary Material.

## Ethics Statement

Ethical approval was obtained by the Ethics Committee of Taipei Hospital, Ministry of Health and Welfare (TH-IRB-0020-0002).

## Author Contributions

H-YC and W-LH investigated and supervised the findings of this work. C-HC and C-YH collected the data and analysis. WC conceived the present idea, wrote the manuscript, and takes primary responsibility for communication with the journal and editorial office during the submission process, throughout peer review and during publication.

### Conflict of Interest

The authors declare that the research was conducted in the absence of any commercial or financial relationships that could be construed as a potential conflict of interest.
