# Supplementary material for: Children Conceived by Assisted Reproductive Technology Prone to Low Birth Weight, Preterm Birth, and Birth Defects: A Cohort Review of More Than 50,000 Live Births During 2011–2017 in Taiwan
Source: Front Pediatr. 2020 Mar 13;8:87. doi: 10.3389/fped.2020.00087 (PMC7082315; doi:10.3389/fped.2020.00087)
Supplement: Supplementary file 1 [file Data_Sheet_1.PDF]

Supplement

Supplement Figure 1. Percentage of births by ART from 2011-2017

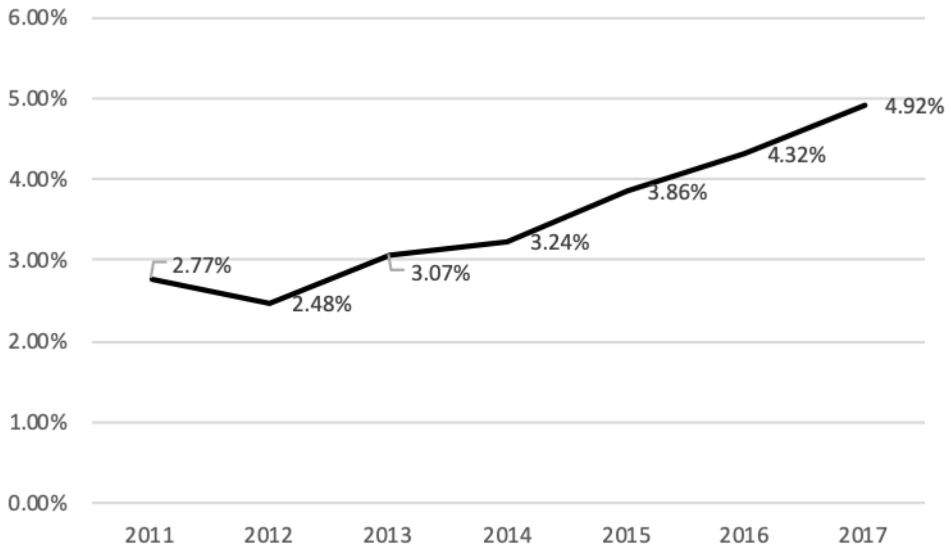

Supplement Figure 2. Percentages of singletons and twins in all pregnancy live cycles of ART conception from 2011-2017. The percentage of twin pregnancy is higher in ART conception than that in natural contraception, but the rates of twin birth in ART decrease slightly during the study period.

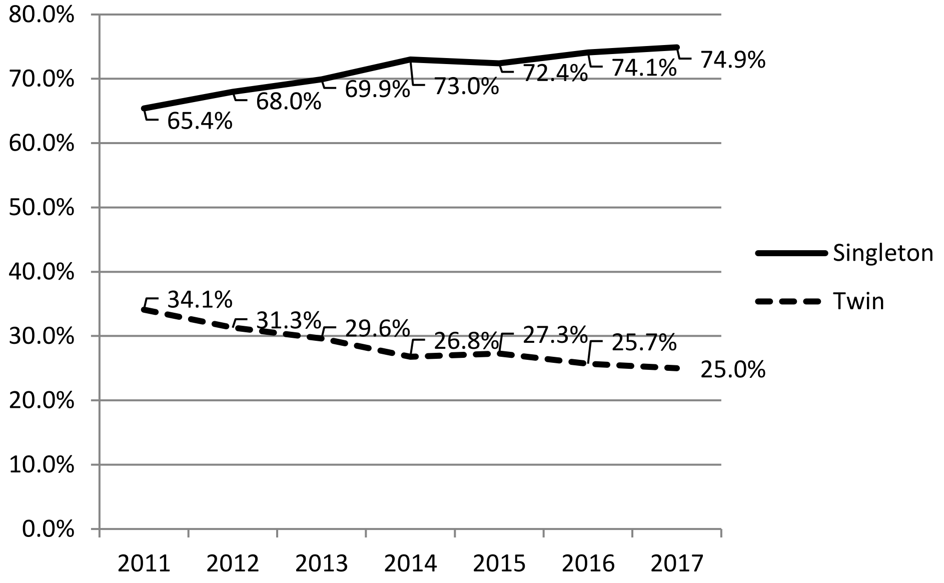

Supplement table 1. Annual proportions of VLBW and LBW in Natural pregnancy and ART conception and ART conception singleton in 2011-2017

	VLBW (< 1500g)			LBW(1500-2499g)	
Time	Natural pregnancy	ART conception	Time	Natural pregnancy	ART conception
2011	0.79%	4.78%	2011	6.49%	36.73%
2012	0.73%	4.52%	2012	6.88%	35.23%
2013	0.73%	4.79%	2013	6.93%	33.68%
2014	0.75%	4.33%	2014	6.90%	30.82%
2015	0.77%	4.40%	2015	7.20%	31.26%
2016	0.81%	3.58%	2016	7.41%	30.80%
2017	0.77%	4.12%	2017	7.05%	32.09%
Time	Natural pregnancy	ART conception singleton	Time	Natural pregnancy	ART conception singleton
2011	0.79%	2.30%	2011	6.49%	9.70%
2012	0.73%	1.90%	2012	6.88%	9.70%
2013	0.73%	2.30%	2013	6.93%	10.00%
2014	0.75%	1.80%	2014	6.90%	9.40%
2015	0.77%	1.90%	2015	7.20%	9.30%
2016	0.81%	1.80%	2016	7.41%	9.60%
2017	0.77%	1.9%	2017	7.05%	9.0%

Supplement table 2. Annual proportions of preterm birth (< 37 weeks) in natural pregnancy, ART conception and ART conception singleton in 2014-2016

	2014			2015			2016		
Maternal age	Natural pregnancy	ART conception	ART conception singleton	Natural pregnancy	ART conception	ART conception singleton	Natural pregnancy	ART conception	ART conception singleton
≦24	8.03%	53.33%	12.5%	8.47%	54.55%	13.3%	8.44%	41.30%	20.7%
25-34	7.56%	34.81%	10.8%	7.68%	38.30%	13.0%	7.91%	37.18%	10.2%
≧35	10.06%	33.36%	13.3%	10.16%	35.20%	13.8%	11.03%	31.95%	13.9%

Supplement table 3. Annual proportions of any defects in Natural pregnancy and ART conception in 2014-2016

	Natural pregnancy	ART conception
Time	**Any defects**	
2014	0.49%	0.58%
2015	0.47%	0.85%
2016	0.30%	1.23%

Supplement table 4. Annual proportions of cardiovascular, nervous, urinary and digestive defects in Natural pregnancy and ART conception in 2014-2016

	Natural pregnancy	ART conception	Natural pregnancy	ART conception	Natural pregnancy	ART conception	Natural pregnancy	ART conception
Time	Cardiovascular Defect		Nervous Defect		Urinary Defect		Digestive Defect	
2014	0.08%	0.19%	0.02%	0.03%	0.06%	0.03%	0.05%	0.07%
2015	0.06%	0.22%	0.01%	0.10%	0.05%	0.07%	0.04%	0.13%
2016	0.04%	0.35%	0.005%	0.06%	0.04%	0.13%	0.02%	0.07%
